# Neurocognitive Safety of Endoscopic Colloid Cyst Resection: Paired Pre- and Post-Operative Cognitive Function from an Exploratory Cohort

**DOI:** 10.3390/cancers17030416

**Published:** 2025-01-27

**Authors:** Umberto Tosi, Amanda Sacks-Zimmerman, Francis Michael Villamater, Jessica S. Spat-Lemus, Kenneth Perrine, Mark Souweidane, Heidi Allison Bender

**Affiliations:** 1 Department of Neurological Surgery, Weill Cornell Medicine, New York, NY 10065, USA; 2 Department of Neurological Surgery, Memorial Sloan Kettering Cancer Center, New York, NY 10065, USA; 3 Department of Psychology, Montclair State University, Montclair, NJ 07043, USA

**Keywords:** colloid cyst, cognition, endoscopy, third ventricular cyst

## Abstract

Objective: Resection of colloid cysts, a rare third ventricle pathology, is accepted clinical practice. Owing to their location proximal to deep gray nuclei and forniceal columns, colloid cysts have been theorized to contribute to cognitive decline. Comprehensive pre- and post-operative cognitive testing, however, has rarely been implemented. Methods: We analyzed formal neuropsychological testing performed in 20 patients undergoing endoscopic cyst resection. Pre- and post-operative performance was compared either for each individual patient or according to aggregated neuropsychological factor scores grouped via expert census. A change in performance was deemed significant if (i) it reached statistical significance and (ii) was greater than 1.5 pre-operative standard deviations. Results: Twenty patients with colloid cysts (average diameter 13.3 ± 1.3 mm) underwent matched pre- and post-operative testing. No patient had a significant change in cognitive performance. Neurocognitive metrics assessing cognitive functions typically subsumed by the temporal (*p* = 0.35), extratemporal (*p* = 0.20), occipitoparietal (*p* = 0.31), or frontal lobes (*p* = 0.11) did not change post-operatively. Similarly, no differences emerged when factor scores were generated according to composite scores of different neurocognitive domains: attention (*p* = 0.32), executive function (*p* = 0.14), language (*p* = 0.98), and visuospatial function (*p* = 0.42). Conclusions: Neuropsychological testing allows for the careful monitoring of cognitive status before and after surgery and for the identification of patients who may benefit from pre- and post-operative cognitive rehabilitation. It should also be used as a valuable surgical psychometric marker and adjuvant. No significant cognitive decline was observed in this cohort.

## Simple Summary

Colloid cysts are a rare pathology. Albeit histologically benign entities, colloid cysts of the third ventricle can have fatal consequences and lead to fulminant hydrocephalus and death. Because of the deformation of the forniceal columns and ventricular enlargement often found at presentation, untreated colloid cysts may lead to neurocognitive deterioration. The effects of surgical resection on cognition, however, remain poorly understood. In this study, we perform an extensive battery of neurocognitive studies longitudinally, pre- and post-operative, in 20 patients undergoing surgical resection of colloid cysts. We demonstrate how surgery is well tolerated with few neurocognitive sequelae.

## 1. Introduction

Colloid cysts are a rare pathology that arise in the third ventricle. Although histologically benign entities, growth in a confined environment usually leads to significant symptomatology, e.g., hydrocephalus contributing to deformation of forniceal fibers, both associated with cognitive decline [1,2,3].

Historically, colloid cysts were removed via an open craniotomy; however, endoscopic resection has now risen to prominence as an accepted surgical modality. While large comparative studies are still needed, endoscopy is believed to be comparable to open surgery with respect to the extent of resection, complication profile, and possibly having a shorter length of stay [4,5]. While generally safe, endoscopic cyst resection still has a steep learning curve, owing in part to the small operative channels intrinsic to a ventriculoscope and to the rarity of colloid cysts, which renders extensive surgical experience difficult to achieve [6,7,8]. Importantly, asymptomatic cysts represent a unique opportunity for intervention, as surgery, if uncomplicated, may avoid the development of any cognitive decline or the development of ‘fulminant hydrocephalus’, often seen in patients who present later on during their disease course [9,10,11,12]. Some now advocate for endoscopy as a first treatment modality for a variety of cysts, both symptomatic and asymptomatic, as stereotactic frameless navigation and pre-operative high-resolution imaging facilitate surgical resection, thus expanding the realm of cysts that are amenable to endoscopic removal [13]. The use of surgery in the asymptomatic, normoventricular patient remains, however, a matter of discussion.

This developing body of surgical expertise, however, has not been paralleled by an equal understanding of the cognitive effects of endoscopic resection. Careful analysis of pre- and post-operative neurocognitive functioning has been presented only in a handful of patients, in part because, in most medical centers, colloid cyst resection is reserved for emergent cases of patients presenting in extremis. A few studies performed in small cohorts often favor patients with symptomatic cysts associated with hydrocephalus [14,15,16]. In this study, one of the largest to our knowledge, we perform a detailed longitudinal paired analysis of pre- and post-operative cognitive function in 20 patients who underwent endoscopic cyst resection to understand the effects that endoscopic surgery had on cognitive functioning and to further develop a framework for the use of comprehensive neurocognitive testing as a valuable surgical adjuvant, to identify patients at risk for a suboptimal outcome, and create pre- and post-operative strategies for both psychoeducation and cognitive remediation. Understanding the cognitive outcomes facilitates understanding the risks of endoscopic colloid cyst resection, which is essential to properly counsel patients—especially asymptomatic ones—on the risks of surgery. Albeit benign histological entities, colloid cysts (and the development of associated hydrocephalus) may lead to devastating cognitive sequelae, which can be prevented with prompt surgical intervention.

## 2. Materials and Methods

### 2.1. Data Collection

This work is a retrospective, single-institution, cohort study of patients treated with endoscopic colloid cyst removal performed at the authors’ home institution. Data were acquired from a prospective registry (Institutional Review Board registry 1706018315) dating back from September 1996 to January 2023, which, given its retrospective nature, did not require informed consent to be obtained. These data were collected in a secured electronic health information management tool (REDCap—supported in part by the National Center for Advancing Translational Science (NCATS) of the National Institute of Health (NIH) Under Award Number UL1TR002384) (project-redcap.org). Patients were chosen from the surgical database if they had cognitive testing performed in English before and after surgical resection.

The data extracted from the medical records included demographic information at the time of surgery (age, gender), presenting symptomatology, side of intervention, surgical approach (transforaminal vs. transseptal), presence of ventriculomegaly on imaging (intended as a frontal–occipital horn ratio (FOR) > 0.37), use of pre-operative CSF diversion, surgical complications (need for conversion to an open craniotomy, post-operative seizure, surgical site infection, intracranial hemorrhage or ischemia, and pulmonary or cardiac event), and need for post-operative CSF diversion. Continuous variables are described as mean ± standard error of the mean (SEM) unless otherwise stated.

### 2.2. Neurocognitive Factors

As part of their clinical course, patients underwent a battery of neuropsychological tests aimed at assessing different domains of cognitive functioning. Not all patients underwent all tests due to external factors, such as session scheduling. Each test yielded one or more variables used in this analysis. A consensus decision from a panel of board-certified neuropsychologists was polled to create factor scores based on the putative neuroanatomical structures that subsume the cognitive test (i.e., temporal vs. extratemporal, and temporal vs. frontal vs. occipitoparietal), as well as the construct the tests are believed to assess (i.e., attention vs. executive function vs. language, vs. memory, vs. visuospatial abilities), consistent with the literature [17]. A detailed description of the specific tests included in each group is provided in Supplementary Materials (Supporting Table S1). For each individual subtest, pre- and post-operative raw scores were transformed into a common metric (z-score) based on published, age-adjusted (and education, if appropriate and applicable) standardization samples. Data are shown as box and violin plots where each dot represents a patient’s performance on a specific test assigned to a given category.

### 2.3. Surgical Procedure

Endoscopic surgical resection of colloid cysts was carried out as previously described [9,18,19]. All procedures were performed by one of the senior authors (MMS) with intraoperative, frame-based surgical navigation. Briefly, following anesthesia induction, the patient is registered in a neutral position. A small burr-hole craniotomy is turned at an entry point defined with surgical navigation. Following dural opening and corticectomy, the endoscope is advanced, following navigation into the lateral ventricle. In cases of small or normal ventricular caliber, the ventricle is first cannulated with a ventriculostomy catheter; gentle fluid insufflation is performed; and finally, the ventriculostomy catheter is removed and the ventriculoscope advanced through the tract thus created.

**Figure 1:**
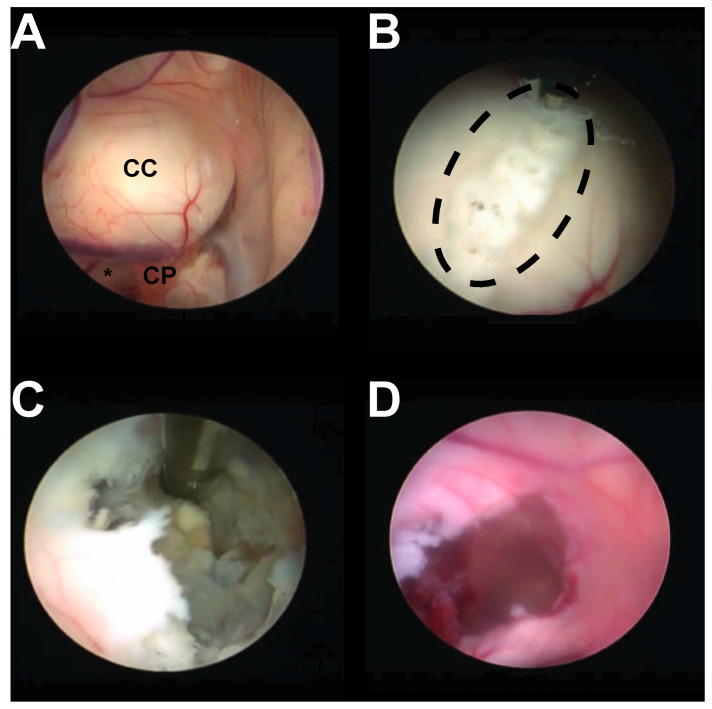
Intraoperative finding. (**A**) Intraoperative image showing a large protruding mass behind the septum pellucidum (CC); notably, the foramen of Monro (asterisk) is normal sized; choroid plexus (CP) is also appreciated. (**B**) Following cyst identification, a septostomy (dashed line) is performed with bipolar cautery. (**C**) Once the cyst is accessed, cyst contents are removed in a stepwise fashion. (**D**) Following cyst resection, the cavity is inspected to confirm homeostasis.

Once in the lateral ventricle, normal anatomy is appreciated, the foramen of Monro is identified, and the cyst location is appreciated (Figure 1). Generally, the cyst capsule is separated from the overlying choroid, and adhesions are coagulated. The cyst contents are decompressed with a combination of cautery, suction aspiration, or tissue shaving devices (available in the most recent cases only); the cyst capsule is then removed with forceps. Hemostasis is achieved with gentle irrigation; at times, a ventriculostomy catheter is left in the ventricle and removed post-operatively in the intensive care unit to enhance hemostasis. In cases in which the cyst is superiorly recessed and not evident at the foramen, a small septostomy is performed as previously described, and the cyst variant is removed in such a fashion [13].

### 2.4. Statistical Analysis

Descriptive statistics were used to characterize the study sample with respect to demographic and clinical factors of interest. Performance across a single domain was gauged as the difference between pre-and post-operative performance, as determined via a parametric, paired *t*-test. The performance of each individual patient was analyzed by comparing pre- and post-operative performance via a parametric, paired t-test. Clinically significant difference was defined as (i) the change reached statistical significance (i.e., *p* < 0.05) and (ii) the change was greater than 1.5 times the standard deviation of the pre-operative performance distribution, as established in the literature [20]. Contingency tables were analyzed via Fisher’s exact test. When the influence of laterality was assessed across domains, a two-way analysis of variance (ANOVA) with repeated measures across pre- and post-operative performance was carried out. All *p*-values are two-sided, with statistical significance evaluated at the 0.05 alpha level. All analyses were performed in GraphPad Prism version 10 (GraphPad Software, San Diego, CA, USA).

## 3. Results

### 3.1. Cohort Characteristics

A total of 20 patients who underwent endoscopic resection of a colloid cyst between 2009 and 2019, for whom pre- and post-operative neurocognitive testing was administered, were extracted from the dataset and included in this analysis. Detailed cohort characteristics are presented in Table 1. The average age was 43.4 ± 2.6 years; 11 (55%) were female. Eight patients (40%) had symptoms at presentation to neurosurgical consultation (subjective cognitive changes, headaches, visual changes, nausea, or vomiting); the rest of the cohort was asymptomatic, with incidentally found cysts when imaging was performed following trauma or for symptoms not obviously related to their cyst (e.g., long-standing history of migraines with remote negative imaging). The average frontal–occipital horn ratio (FOR), as a measure of ventricular size, was 0.36 ± 0.01; seven patients had ventriculomegaly on presentation (average FOR 0.42 ± 0.01). The average greatest diameter of the cyst was 13.2 ± 1.3 mm. One patient had prior resection. One patient presented in extremis requiring pre-operative EVD placement and underwent pre-operative testing following initial recovery while in the hospital. Seven patients (35%) underwent surgery via a left-sided approach; four (20%) had a transseptal cyst resection. One patient required conversion to open craniotomy for surgery and, along with another patient, required post-operative CSF diversion. No other complication occurred; no cyst recurrence was observed during clinical follow-up, which was, on average, 30.9 ± 6.1 months from surgery. Even in cases of longer follow-up (as long as 101 months), no long-term sequelae were observed. Tests were performed, on average, 31.4 ± 11.9 days prior to surgery and 7.0 ± 2.6 months thereafter.

### 3.2. Neuropsychological Test Analysis

A total of 24 neuropsychological tests were administered to the twenty patients; not all tests were administered to all patients (a total of 685 tests were administered to the entire cohort, creating 308 pre- and post-operative pairs). Tests were grouped as described in the Supplementary Materials (Supporting Table S2) following a survey across board-certified neuropsychologists to assess for temporal vs. extratemporal function, temporal vs. frontal vs. occipitoparietal function, and to measure cognitive functioning within the domains of attention, executive functioning, language, memory, and visuospatial functioning.

### 3.3. Individual Performances

**Figure 2:**
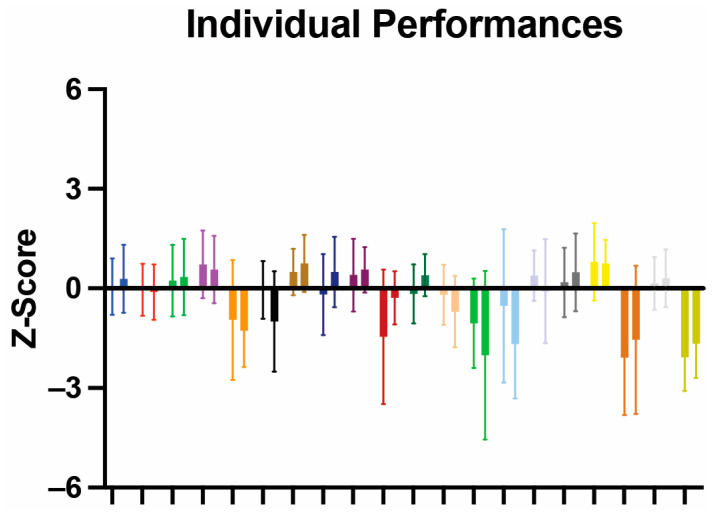
Patient-by-patient performance. Plots showing pre- and post-operative performance across all cognitive tasks for each individual patient. Data shown as mean ± SD.

We first set to establish if, following surgery, there was any change in all cognitive performances for each patient by transforming pre- and post-operative performance into a common metric (z-scores) and evaluating performance across all tests administered, i.e., if any patient had cognitive worsening or improvement. Of the 20 patients studied, none had a significant change in their global pre-operative performance (Figure 2). The differences observed between pre- and post-operative performances did not reach clinical significance (i.e., they were never both statistically significant and greater than 1.5 times the pre-operative standard deviation). Only one patient had a pre-operative clinically significant difference from the average population (i.e., z-score inferior to 0), which was maintained post-operatively. However, they did not experience any overall change between pre- and post-operative performance. Of importance, no difference was observed between pre- and post-operative performance for three patients with a complicated clinical course. More specifically, one patient presented in extremis, requiring pre-operative CSF diversion in the form of ventriculostomy; one required conversion to open craniotomy because of significant intraoperative bleeding, followed by ventriculoperitoneal shunting; and a third patient required post-operative ventriculoperitoneal shunting.

Individual patient’s neuropsychological test results were then stratified by neuroanatomical or neurocognitive domains to increase power and reduce type 1 errors; a total of nine domains were established (as described in the Supplementary Materials), with each patient being tested across all domains (thus creating 180 pre–post-operative domain pairs) (Supporting Figure S1). A clinically significant difference was observed only for one patient in the memory domain (1/180, 0.56%); the patient experienced worsening in memory function following cyst resection. Of note, this patient had the largest cyst in this cohort (27 mm) and the second largest ventricular caliber (FOR of 0.44). The patient was nonetheless followed clinically and, approximately six months following initial post-operative testing (carried out three months post-resection), reported significant subjective improvement with a full return to work.

Subjectively, no patient reported a notable cognitive deterioration post-operatively, with five patients reporting subjective improvement (however, not appreciated in neurocognitive testing); 17 (85%) patients returned to work post-operatively; of the three patients who did not return to work, two had not been working for years before surgery, and one patient was the one presenting in extremis.

### 3.4. Anatomical Function (Frontal vs. Temporal vs. Occipitoparietal)

**Figure 3:**
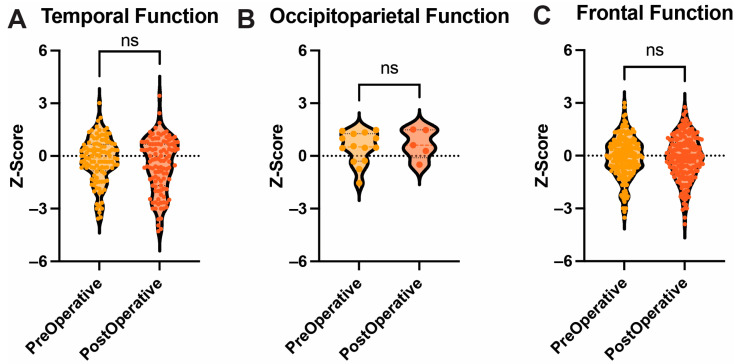
Anatomical Function. Box and Violin plots showing no difference between pre- and postoperative performance in temporal (**A**), occipitoparietal (**B**), and frontal (**C**) functions. Ns = not significant.

When neurocognitive tests were grouped based on cerebral lobe localization targets, seven variables assessed temporal function, two assessed occipitoparietal function, and 15 tested frontal function. No difference was observed for temporal function (*p* = 0.35). Similarly, no difference was observed on analysis of occipitoparietal (*p* = 0.31) and frontal (*p* = 0.11) functions between pre- and post-operative assessments (Figure 3). Similar results were found when the extratemporal function was assessed pre-operatively (228 variables) and post-operatively (222 variables) (*p* = 0.20) (Supporting Figure S2).

### 3.5. Specific Cognitive Functions (Attention, Executive Functioning, Language, and Memory)

**Figure 4:**
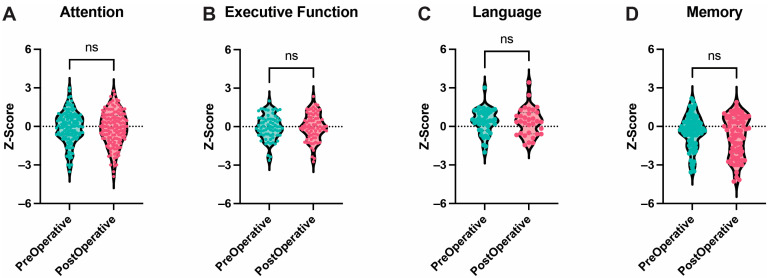
Higher Cognitive Functions. Box and Violin plots showing no difference between pre- and post-operative performance in attention (**A**), executive function (**B**), language (**C**), and memory (**D**). Ns = not significant.

No statistically significant difference was found between pre- and post-operative attention across nine variables (*p* = 0.32), executive function (six variables, *p* = 0.14), language (three variables, *p* = 0.98), memory (five variables, *p* = 0.42), and visuospatial function (one variable only) (Figure 4).

### 3.6. Lateralization of Approach

**Figure 5:**
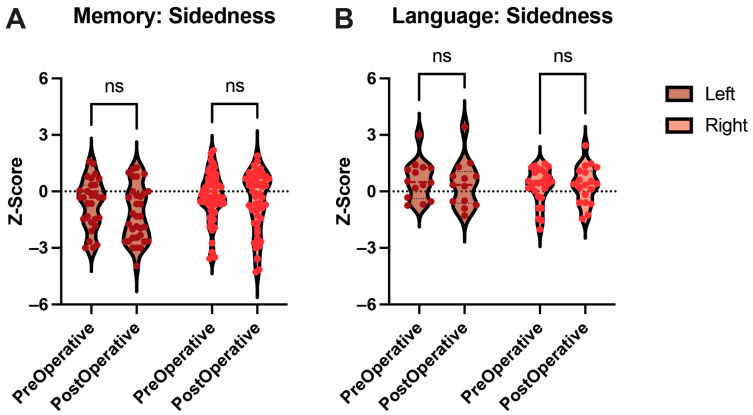
Sidedness of Memory and Language. Box and Violin plots showing no difference between pre- and post-operative performance regardless of the sidedness of approach for both memory (**A**) and language (**B**) functions. Ns = not significant.

The data were then grouped based on the laterality of the approach: seven patients underwent a left-sided approach, and 13 underwent a right-sided approach. Memory function was compared across the two groups. No statistically significant difference was found between pre- and post-operative function with either a left-sided approach (*p* = 0.81) or with a right one (*p* = 0.27). Similar results were observed when language function was assessed, with no statistically significant difference observed between pre- and postoperative function with either a left-sided approach (*p* = 0.66) or a right-sided one (*p* = 0.91) (Figure 5).

## 4. Discussion

In this study, we have shown effective utilization of extensive pre- and post-operative cognitive testing in patients undergoing endoscopic resections of colloid cysts via a multidisciplinary, collaborative team of neurosurgeons and neuropsychologists. Our study relied on a consensus of experts to create neuropsychological factor scores. We recognize how a factor analytic approach (e.g., varimax rotation) could be performed, though a larger sample size would be necessary and will be the subject of further studies [21]. Our analysis, however, included examining patients’ performance relative to demographically based normative samples as well as patients’ performance relative to their baseline metrics. This approach is superior to subjective reports; however, our data are concordant with subjective reporting and return to work status. The potential benefits of our approach include the ability to guide pre-operative discussions with respect to cognitive prognostication in patients undergoing resection and the capacity to define the risks of post-operative neurocognitive morbidity, with implications for informed consent, psychoeducation, and post-operative treatment or rehabilitation planning. The methods highlighted in this study should provide a framework for the continuity of care for colloid cyst patients, as it is becoming commonplace at our institution for these patients to undergo a standard neuropsychological battery both pre- and post-operatively, in light of the protocol and data here shown [22,23,24,25]. Importantly, this work was made possible thanks to continuous close collaboration in a multidisciplinary team, with protocols in place for expedited patient referral to neuropsychological testing and numerous multidisciplinary discussions to ascertain the optimal therapeutic strategy and its effects on cognition.

It is notably difficult to separate possible changes in post-operative function from the natural history of colloid cysts, as cognitive testing has not been compared longitudinally on age-matched cohorts that have not undergone surgery or to compare our surgical approach (endoscopy) with open craniotomy for resection, which is routinely performed but again poorly followed from a neuropsychological standpoint, in part owing to the rarity of colloid cysts [26]. For instance, one patient in our cohort performed significantly below the normative mean pre-operatively, possibly relating to their disease course and specific manifestation of cyst pathology (i.e., large cyst with ventriculomegaly). Studies that have assessed the effects of surgery (or watchful waiting) often lacked extensive pre- and postoperative testing, such as the current neurocognitive battery of measures utilized here [15,27]. Overall, these findings provide further evidence for the cognitive tolerability of the endoscopic approach to colloid cyst resection.

Compared to open surgery, ventriculoscopy—and neuroendoscopy in general—is believed to be better tolerated owing, at least in part, to decreased tissue destruction and retraction—the endoscopic path being a fraction of that required by open surgery—at the cost of decreased exposure. Albeit direct neurocognitive comparisons between open and endoscopic resection of colloid cysts remain to be performed in large longitudinal cohorts, other studies have shown, at least in part, the tolerability of this procedure [1]. Other studies that have assessed outcomes following endoscopic (or endoscopic-assisted) resection of skull base pathologies have found this surgical approach to achieve similar results to their microsurgical counterparts, with potentially fewer complications—again owing to smaller surgical corridors and a possibly more benign neurocognitive profile [28,29,30,31]. Of course, this has to be balanced against the steep learning curve observed in endoscopic series—the utilization of rigorous neuropsychological testing will allow for careful balancing of surgical goals with improved understanding of post-operative cognitive outcomes [32].

The optimal timing to perform extensive neuropsychological testing remains a matter of debate. In this study, post-operative testing was performed 7.0 ± 2.6 months after surgery. On the one hand, cognitive function is expected to somewhat improve further from neurosurgery due to factors related to neuroplasticity, diminished inflammation, and physiological healing factors. However, with exceeding amounts of time, other confounding variables may contribute to neurocognitive changes (e.g., aging or other comorbidities). In the neurosurgical epilepsy literature, where cognitive testing is routinely performed, experts appreciate longitudinal changes post-operatively [33,34,35]. A longitudinal perspective of neurocognitive functioning in this population may be essential in order to track various trajectories of neurocognitive recovery, as the only patient who had experienced worsening in her memory function recovered in six months, indicating the tolerability of this procedure.

Furthermore, in our study, our data showed no difference in pre- and post-operative performance in memory and language function when a left-sided approach was compared with a right-sided one. The cognitive effects of a left-sided endoscopic approach have long been debated, with some fearing a theoretical worsening in verbal memory functioning due to the lateralization of cognitive functions, similar to literature on resective surgery for epilepsy [36,37]. Due to the small and targeted path offered by the endoscopic approach when compared to open surgery, regardless of the laterization of the approach, one can avoid most cognitively eloquent structures [11].

Similarly, forniceal manipulation and injury have been associated with cognitive decline, particularly within the area of memory-related functioning, with numerous studies associating forniceal damage on imaging following traumatic brain injury with subsequent amnesia development [38,39,40]. We have recently described a transseptal intraforniceal approach to superiorly recessed colloid cysts that relies on a septostomy and forniceal manipulation for cyst removal [13]. One of the theoretical risks of this approach is the development of subsequent cognitive decline, albeit not clearly observed in our original cohort. In the cohort described in this study, a transseptal approach was *not* associated with cognitive worsening, again indicating the neurocognitive safety of this surgical corridor (Supporting Figure S3). Given the rarity of colloid cysts requiring a septostomy for resection, further studies are needed to ultimately determine the safety profile of this type of surgery.

These data are further helpful in guiding pre-operative psychoeducation with patients. In our experience, most patients present with incidentally found colloid cysts. Given the proven safety of this approach and our study contributing to the literature on cognitive safety, which achieves high rates of gross total resections without recurrence or significant complications, elective surgery is being more frequently offered to patients with incidental colloid cysts [41,42,43]. Although no difference was seen in our cohort between patients with and without ventriculomegaly (Supporting Figure S4), its effect on cognition has been well-described [44]. The data presented here, which show the low morbidity from the cognitive perspective, supports elective surgical resection utilizing the endoscopic technique, albeit further studies are needed to confirm these findings. Moreover, it provides insight into a new clinical workflow and infrastructure, whereby patients deemed at risk can be counseled from a multi-disciplinary perspective about the risks of the procedure or enrolled in the beginning phases of their clinical course in pre- or postoperative rehabilitation programs, as performed for other neurosurgical pathologies (e.g., epilepsy) [45,46].

Given the fact that there is mounting evidence to suggest that formal neurocognitive testing may be necessary to detect small but significant cognitive changes and can assist a clinician’s decision-making, this multidisciplinary practice where neuropsychologists are involved from early stages of patient care is becoming commonplace in our institution, with longer follow-up and longitudinal testing being performed to capture more final cognitive endpoints. As it is being integrated into clinical practice, it will allow for the determination of safety and neurocognitive outcomes of a wide array of neurosurgical procedures, increasing clarity in the context of pre-operative psychoeducation.

### Limitations

Our study was limited by its small sample size and retrospective nature. Given that this cohort in which cognitive data were available represents a small portion of our decade-long surgical experience, only adult patients (between 23- and 63-year-old) are included; our surgical experience, however, is not limited to this age group, with pediatric and senior patients operated on as well. Future studies will address these demographics. As for all studies relying on neurocognitive testing, practice effect—the process by which patients perform better on a test the second time due to prior exposure—has to be considered. This effect was mitigated, in part, by defining clinical significance as a combination of statistical significance and change greater than 1.5 standard deviations. Other methods, relying on reliable-change indices (where a patient’s performance is compared, through various mathematical methods, to age- and repetition-adjusted standards), which could not be readily applied to this database, are also being developed as part of our continuous efforts. To further mitigate repetition effects, our data were normalized against age-adjusted norms; we further defined a “clinically significant change” as including both statistical significance and a difference greater than 1.5 times the pre-operative standard deviation to minimize practice effect further [20]. Future studies may also rely on reliable change indices. Finally, our study may be affected by selection bias, as only 20 patients (out of the 110 that underwent resection between 2009 and 2019) underwent neurocognitive testing: some patients presented in extremis; some refused; and for others, other factors came into play (e.g., lack of insurance coverage) [47]. Future studies, however, will need a larger cohort and, possibly, age-matched control groups to account for these effects.

## 5. Conclusions

In a small cohort of patients, endoscopic resection of colloid cysts is safe and does not result in any cognitive worsening, as demonstrated by extensive pre- and post-operative neurocognitive testing. Given this safety, which needs redemonstrations with further studies, this procedure should be offered electively, and cognitive testing performed to best identify patients at risk of deterioration if this were to occur.

## Figures and Tables

**Table 1:** Pre- and post-operative cohort characteristics.

	N (%)
Patients	20 (100)
Female Gender	11 (55)
Age (yrs)	43.4 ± 2.6
Symptomatic	8 (40)
Length of Follow-up (mo)	30.9 ± 66.1
FOR	0.36 ± 0.01
FOR > 0.38	7 (35)
Maximal Cyst Diameter	13.2 ± 1.3
Previous Resection	1 (5)
Gross total resection	19 (95)
Pre-operative EVD	1 (5)
Transforaminal Surgery	4 (20)
Left Sided Approach	7 (35)
Post-operative Complications	1 (5)
Post-operative CSF diversion	2 (10)
Recurrence	0 (0)

## Data Availability

The datasets presented in this article are not readily available because of patients’ privacy concerns.

## Supplementary Materials

The following supporting information can be downloaded at https://www.mdpi.com/article/10.3390/cancers17030416/s1. Figure S1. Subtype-specific patient performances. Figure S2. Assessment of Temporal and Extratemporal Function. Figure S3. The Effect of Approach on Memory. Figure S4. The Effects of Ventriculomegaly. Table S1. List of neurocognitive tests performed and how they were clustered. Table S2. Summary of each test and its associated cognitive domain. References [48,49,50,51,52,53,54,55,56,57] are cited in Supplementary Materials.
