# Supplementary material for: Neurocognitive Safety of Endoscopic Colloid Cyst Resection: Paired Pre- and Post-Operative Cognitive Function from an Exploratory Cohort
Source: Cancers (Basel). 2025 Jan 27;17(3):416. doi: 10.3390/cancers17030416 (PMC11815914; doi:10.3390/cancers17030416)
Supplement: Supplementary file 1 [file cancers-17-00416-s001.zip › cancers-3410107-supplementary.pdf]

Supporting Materials to

Neurocognitive Safety of Endoscopic Colloid Cyst Resection: Paired Pre- and Post-Operative Cognitive Function from an Exploratory Cohort

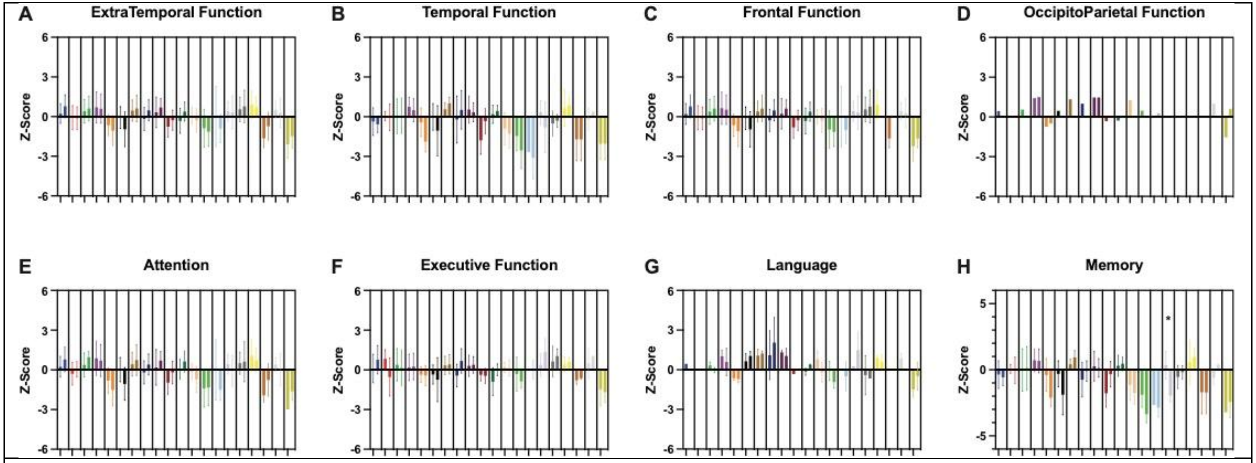

Supporting Figure S1. Subtype-specific patient performances. Plots showing pre- and post-operative performance for each individual patient, subclustered by cognitive task or putative anatomical location: extratemporal (A), temporal (B), frontal (C), occipitoparietal (D), attention (E), executive function (F), language (G), and memory (H).

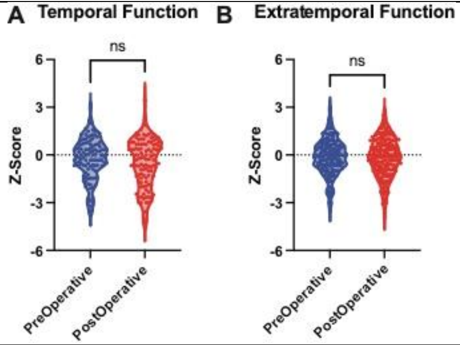

Supporting Figure S2. Assessment of Temporal and Extratemporal Function. Box and Violin plots showing no difference between pre- and postoperative performance in temporal (A) and extratemporal (B) function.

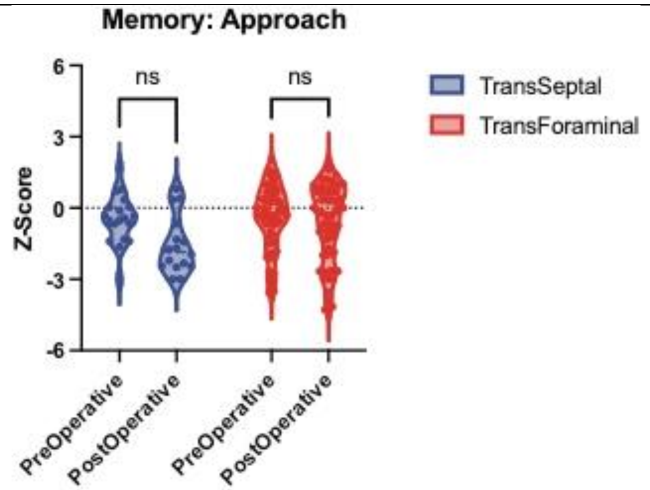

Supporting Figure S3. The Effect of Approach on Memory. Box and Violin plot showing no difference between pre- and post-operative performance regardless of type of approach for memory.

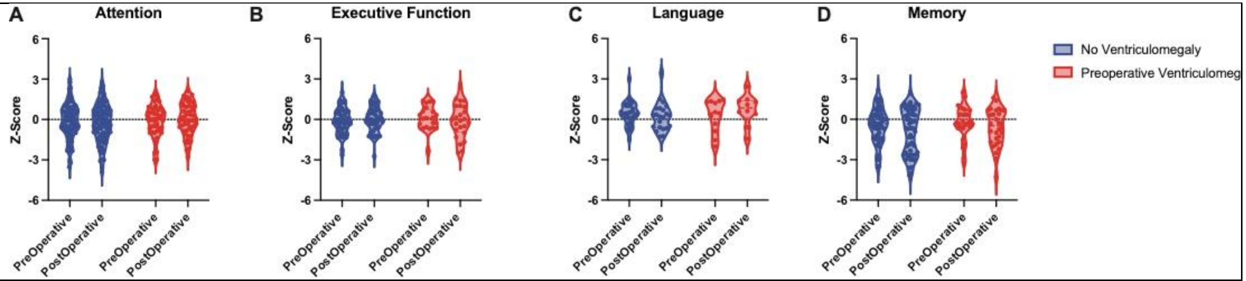

Supporting Figure S4. The Effects of Ventriculomegaly. Box and Violin plot showing no difference between pre- and post-operative performance in patients with and without preoperative ventriculomegaly in attention (A), executive function (B), language (C), and memory (D).

Neuropsychological Test	Abbreviation	Description	Anatomical Location	Cognitive Factor	Citation
Wechsler Test of Adult Reading[1]	WTAR	Pronouncing 50 irregularly spelled words, presented one at a time.	Occipitoparietal	Language	Wechsler, 2001
Color Trails A[2]	CTT-A	Connecting numbered circles, in ascending order, from 1-25 as quickly as possible.	Frontal	Attention	D'Elia et al., 1996
Color Trails B[2]	CTT-B	Connecting pink and yellow numbered circles, in ascending order, while alternating between colors. Each number is presented twice, once in pink and once in yellow, with one color serving as a distractor.	Frontal	Attention	D'Elia, et al., 1996
Stroop Test – Word[3]	Stroop-W	Reading list of color-words, printed in black ink, as quickly as possible in 45 second trial.	Frontal	Attention	Golden, C. J., 1978
Stroop Test – Color[3]	Stroop-C	Stating the ink colors, printed in patches, as quickly as possible in 45 second trial.	Frontal	Attention	Golden, C. J., 1978
Stroop Test – Color-Word[3]	Stroop-W-C	Stating the color of color-words, printed in incongruently colored ink, as quickly as possible in 45 seconds.	Frontal	Executive function	Golden, C. J., 1978

Wisconsin Card Sorting Test[4]	WCST	Sorting of a set of cards according to implicit rules and limited corrective feedback.	Frontal	Executive function	Grant, D. A., & Berg, E. A., 1948
Rey Auditory Verbal Learning Test: Trial 1[5]	RAVLT-1	Recalling words read to participant from 15-word list (List A) immediately after oral presentation.	Frontal	Attention	Peaker, A., & Stewart, L. E., 1989
Rey Auditory Verbal Learning Test: Trial 5[5]	RAVLT-5	Fifth trial of recalling words read to participant from 15-word list (List A) immediately after oral presentation.	Frontal	Attention	Peaker, A., & Stewart, L. E.,1989
Rey Auditory Verbal Learning Test: Trial B[5]	RAVLT-B	Recalling words read to participant from 15-word interference list (List B) immediately after presentation.	Frontal	Executive function	Peaker, A., & Stewart, L. E., 1989
Rey Auditory Verbal Learning Test: Trial 6[5]	RAVLT-6	Recalling words from List A following completion of interference list (List B) recall.	Temporal	Memory	Peaker, A., & Stewart, L. E.,1989
Rey Auditory Verbal Learning Test: Trial 7[5]	RAVLT-7	Delayed recall of words from List A.	Temporal	Memory	Peaker, A., & Stewart, L. E.,1989
Rey Auditory Verbal Learning Test: Delayed Recognition[5]	RAVLT-DR	Recognition task requiring identification of words from List A.	Temporal	Memory	Peaker, A., & Stewart, L. E.,1989
Wechsler Memory Scale 4th Edition: Logical Memory I[6]	LM I	Recalling an orally presented short story immediately	Frontal	Attention	Wechsler, D., 2009

		after presentation.			
Wechsler Memory Scale 4th Edition: Logical Memory II[6]	LM II	Recalling an orally presented short story following time delay.	Temporal	Memory	Wechsler, D., 2009
Rey Auditory Verbal Learning Test: Trials 1-5[5]	RAVLT-1-5	Recalling words read to participant from 15-word list (List A) immediately after each oral presentation.	Frontal	Attention	Peaker, A., & Stewart, L. E., 1989
Animal Naming[7]	COWAT-N	Stating as many animal names as possible within one minute.	Temporal	Language	Benson et al., 1983
Controlled Oral Word Association Test[7]	COWAT	State as many words starting with a letter	Frontal	Executive Function	Benson et al., 1983
Boston Naming Test[8]	BNT	Naming of objects presented in pictures.	Temporal	Language	Kaplan et al., 1983
Rey Complex Figure Test: Copy[9]	RCFT-C	Copying of a complex geometric figure onto sheet of paper.	Occipitoparietal	Visuospatial	Rey, A., & Osterrieth, P. A., 1941
Rey Complex Figure Test: Delayed Recall[9]	RCFT-DR	Reproducing complex figure after a 30-minute time delay.	Temporal	Memory	Rey, A., & Osterrieth, P. A., 1941
Wechsler Adult Intelligence Scale 4th Edition: Digit Span Forward[10]	DSF	Repetition of series of digits of increasing length.	Frontal	Attention	Wechsler, D. 2008
Wechsler Adult Intelligence Scale 4th Edition: Digit Span Backward[10]	DSB	Repetition of serious of digits of increasing length in reverse order.	Frontal	Executive function	Wechsler, D. 2008
Wechsler Adult Intelligence Scale 4th Edition: Total Score[10]	DST	Total score of the Wechsler repetition tests	Frontal	Executive Function	Wechsler, D. 2008

Supporting Table S1. List of neurocognitive tests performed and how they were clustered.

Neuropsychological Test	Temporal / Extratemporal Function	Anatomical Function	Cognitive Domain
WTAR	Extratemporal	Occipitoparietal	Language
Color Trails A	Extratemporal	Frontal	Attention
Color Trails B	Extratemporal	Frontal	Attention
Stroop Word	Extratemporal	Frontal	Attention
Stroop Color	Extratemporal	Frontal	Attention
Stroop Color / Word	Extratemporal	Frontal	Executive function
WCST	Extratemporal	Frontal	Executive function
RAVLT-1	Extratemporal	Frontal	Attention
RAVLT-5	Extratemporal	Frontal	Attention
RAVLT-B	Extratemporal	Frontal	Executive function
RAVLT-6	Temporal	Temporal	Memory
RAVLT-7	Temporal	Temporal	Memory
RAVLT-DR	Temporal	Temporal	Memory
Logical Memory I	Extratemporal	Frontal	Attention
Logical Memory II	Temporal	Temporal	Memory
RAVLT-1-5	Extratemporal	Frontal	Attention
Animal Naming	Temporal	Temporal	Language
COWAT	Extratemporal	Frontal	Executive Function
Boston Naming	Temporal	Temporal	Language
RCFT-C	Extratemporal	Occipitoparietal	Visuospatial
RCFT-DR	Temporal	Temporal	Memory
DSF	Extratemporal	Frontal	Attention
DSB	Extratemporal	Frontal	Executive function
DST	Extratemporal	Frontal	Executive Function

Supporting Table S2. Summary of each test and its associated cognitive domain.

REFERENCES

1. Wechsler, D. *Wechsler Test of Adult Reading: WTAR*; The Psychological Corporation: San Antonio, TX, USA, 2001.
2. D'Elia, L.F.; Satz, P.; Uchiyama, C.L.; White, T. *Color Trails Test: Professional Manual*; Psychological Assessment Resources: Odessa, FL, USA, 1996
3. Golden, C.J. *Stroop Color and Word Test: A Manual for Clinical and Experimental Uses*; Stoelting Co.: Chicago, IL, USA, 1978.
4. Grant, D.A.; Berg, E.A. *Wisconsin Card Sorting Test*; APA PsycTests: Washington, DC, USA, 1948.
5. Rey, A. *L'examen Clinique en Psychologie [The Clinical Psychological Examination]*; Presses Universitaires de France: Paris, France, 1964.
6. Wechsler, D. *Wechsler Memory Scale—Fourth Edition (WMS-IV)*; APA PsycTests: Washington, DC, USA, 2009.
7. Benton, A.L.; Hamsher, d.S.K.; Sivan, A.B. *Controlled Oral Word Association Test (COWAT)*; APA PsycTests: Washington, DC, USA, 1983.
8. Kaplan, E.; Goodglass, H.; Weintraub, S. *Boston Naming Test (BNT*); APA PsycTests: Washington, DC, USA, 1993.
9. Rey, A.; Osterrieth, P.A. *Rey-Osterrieth Complex Figure Copying Test*; APA PsycTests: Washington, DC, USA, 1941.
10. Wechsler, D. *Wechsler Adult Intelligence Scale--Fourth Edition (WAIS-IV)*; APA PsycTests: Washington, DC, USA, 2008
